# Royal Orthopædic Hospital

**Published:** 1902-05-10

**Authors:** 


### ROYAL ORTHOPÆDIC HOSPITAL.

Messrs. Mellor, Smith and May, writing on behalf of Mr. H. A. Reeves under date of April 30th, request that the following corrections of the report of the proceedings at the annual court, printed in The Hospital of the 19th ult., may be made:—" (1) Mr. Reeves did not, as suggested by your report, say he wanted to hear at once about the grant from King Edward's Fund. What he did say was that he wished to draw the attention of the meeting to the remarks of Mr. Ernest Flower M.P., as recorded in the minutes of last year's annual meeting with reference to the contribution from that Fund. (2) Mr. Reeves did not inquire why Mr. Cuerton had been elected to the vice-presidency. He had not the least objection to Mr. Cuerton's election to that office, and had in fact voted for him in committee. (3) Your report is totally incorrect in stating that Mr. Reeves objected to Mr. Richard Biddulph Martin's advent on the committee, as Mr. Reeves made no such objection. (4) Mr. Reeves did not describe the Council of King Edward's Fund as a parcel of amateurs who knew nothing about medical matters. It would have been absurd for him to suggest anything of the kind seeing that he is of course well aware that there are and have been many eminent members of the medical profession connected with that Fund, and his remark had reference only to certain members of the committee of the Orthopædic Hospital, who, though meaning well by the institution, were, he said, new to hospital affairs."

The annual court lasted two hours, and there was at times a good deal of excitement, making it difficult to hear distinctly, especially when Mr. Reeves was speaking. The shorthand notes taken by our representative would indicate that some at any rate of the four paragraphs set out above, whatever else they may be, cannot with any regard to strict accuracy be properly described as corrections of our report.

